# Supplementary material for: Decursinol Angelate Inhibits Glutamate Dehydrogenase 1 Activity and Induces Intrinsic Apoptosis in MDR-CRC Cells
Source: Cancers (Basel). 2023 Jul 8;15(14):3541. doi: 10.3390/cancers15143541 (PMC10377166; doi:10.3390/cancers15143541)
Supplement: Supplementary file 1 [file cancers-15-03541-s001.zip › cancers-2469817-Supplementary File S1.pdf]

Original blots data

Figure 1 and Figure 2

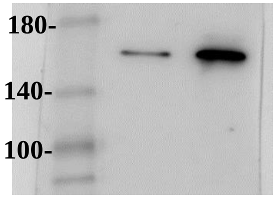

MDR1- 130- 180 kDa

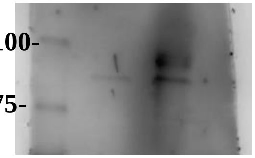

ABCB5- 89.8 kDa

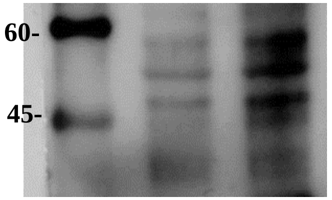

p-ERK1/2 - 44 kDa

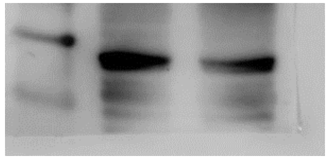

Ki 67

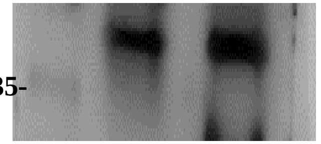

β-actin- 45 kDa

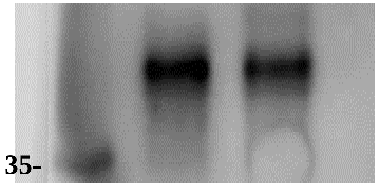

ERCC1- 39 kDa

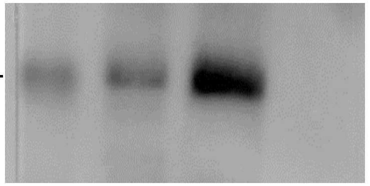

γH2Ax- 15 kDa

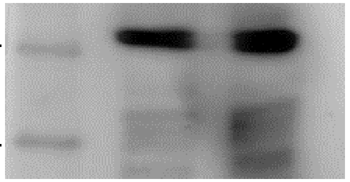

β-actin- 45 kDa

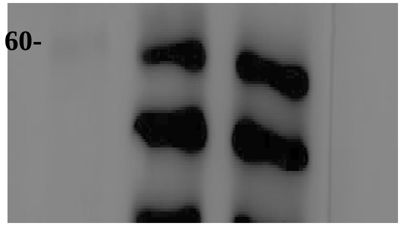

GDH 1/2 – 52 kDa

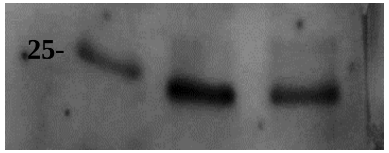

Gpx 1- 23 kDa

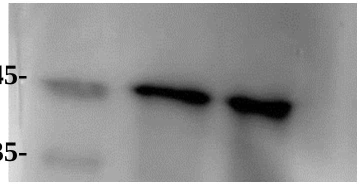

β-actin- 45 kDa

Figure 4

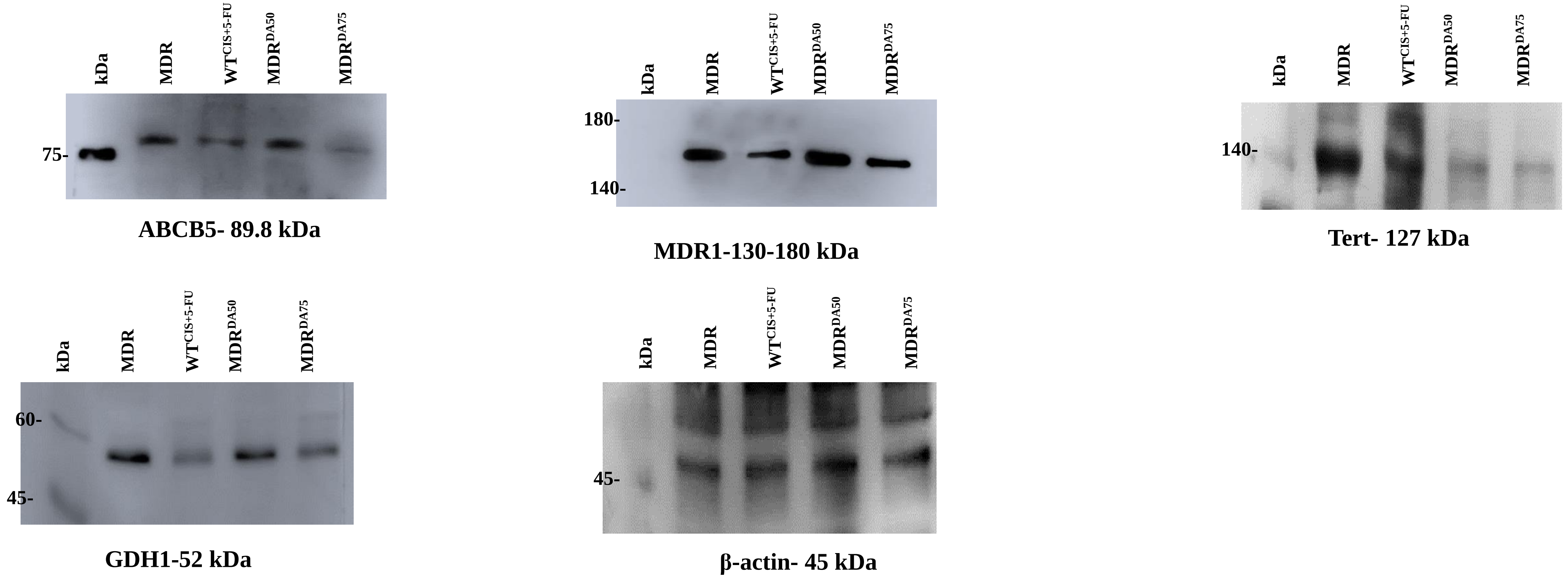

Figure 5

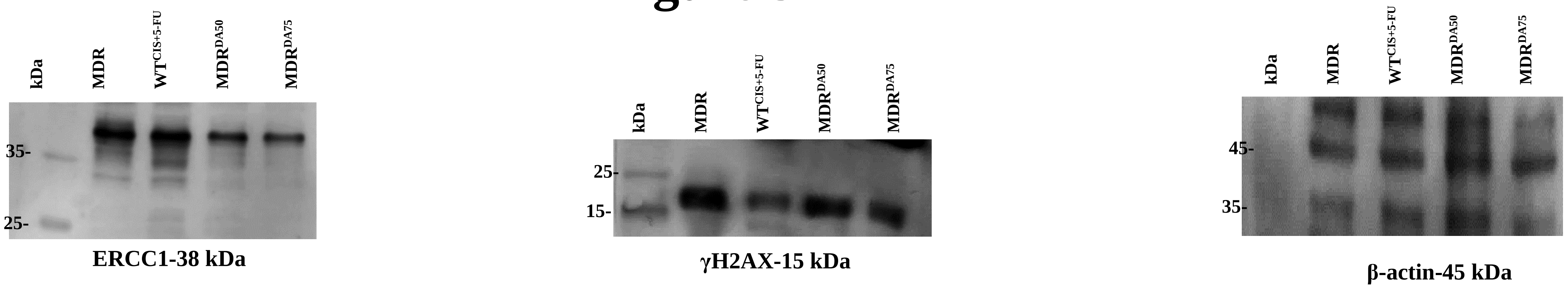

Figure 6

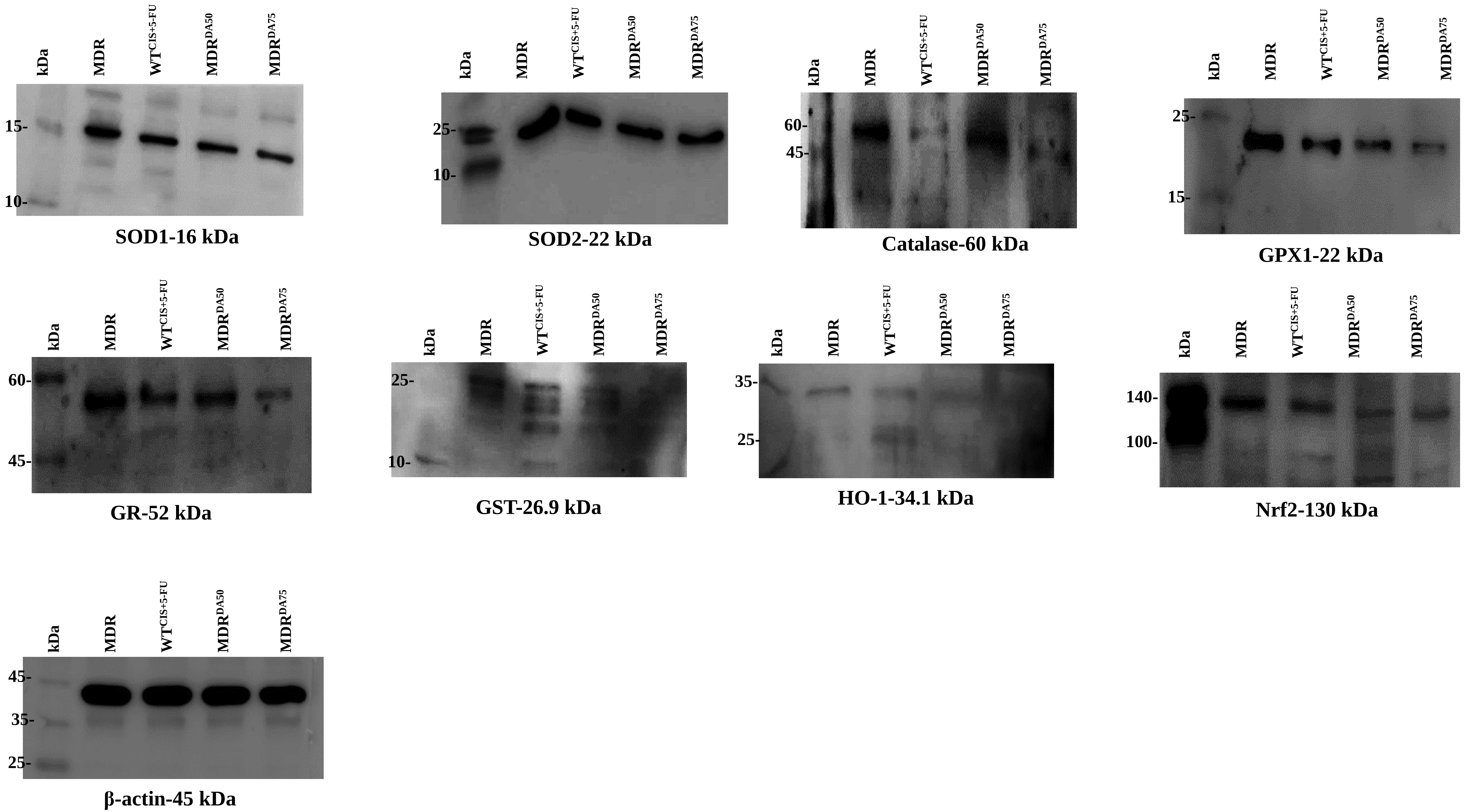

kDa
MDR
WTCIS+5-FU
MDRDA50
MDRDA75
15-
10-
SOD1-16 kDa
25-
10-
SOD2-22 kDa
60-
45-
Catalase-60 kDa
25-
15-
GPX1-22 kDa
60-
45-
GR-52 kDa
25-
10-
GST-26.9 kDa
35-
25-
HO-1-34.1 kDa
140-
100-
Nrf2-130 kDa
45-
35-
25-
β-actin-45 kDa

Figure 7

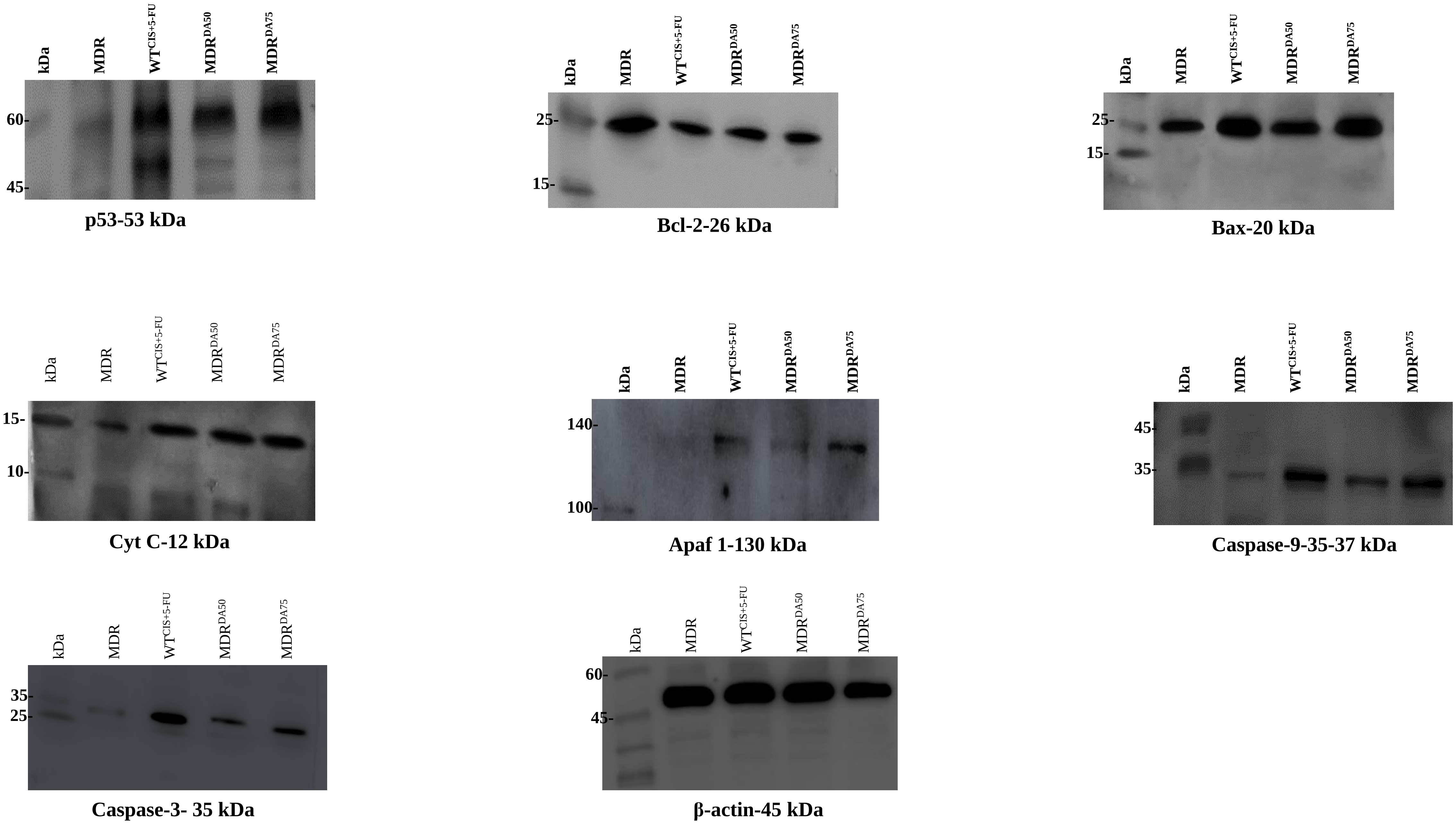
kDa
MDR
WT$^{CIS+5-FU}$
MDR^{DA50}
MDR^{DA75}
60-
45-
p53-53 kDa
25-
15-
Bcl-2-26 kDa
Bax-20 kDa
15-
10-
Cyt C-12 kDa
140-
100-
Apaf 1-130 kDa
45-
35-
Caspase-9-35-37 kDa
35-
25-
Caspase-3- 35 kDa
60-
45-
β-actin-45 kDa

Figure 9

HCT116^{WT}

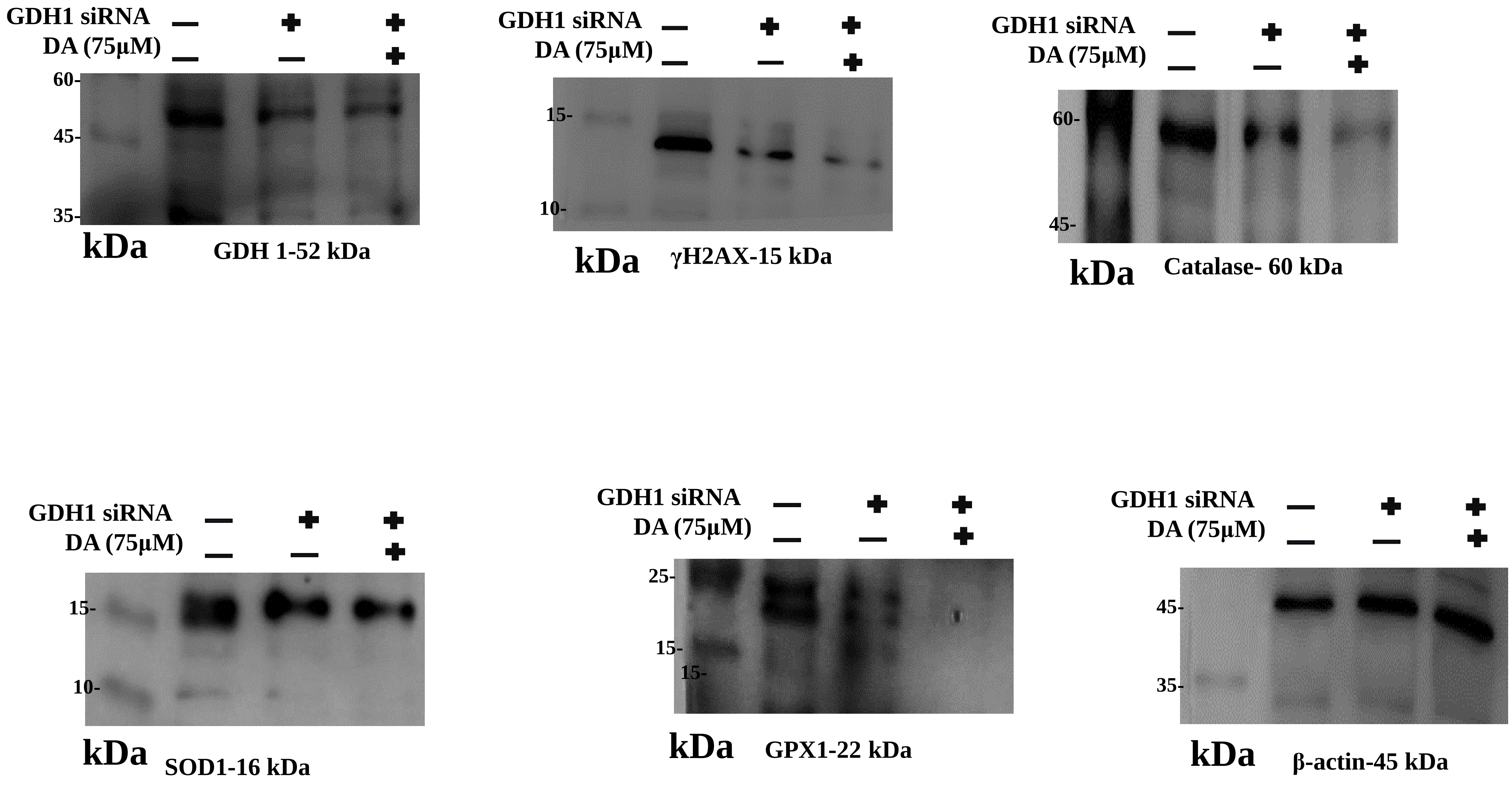

Figure 9

HCT116^{MDR}

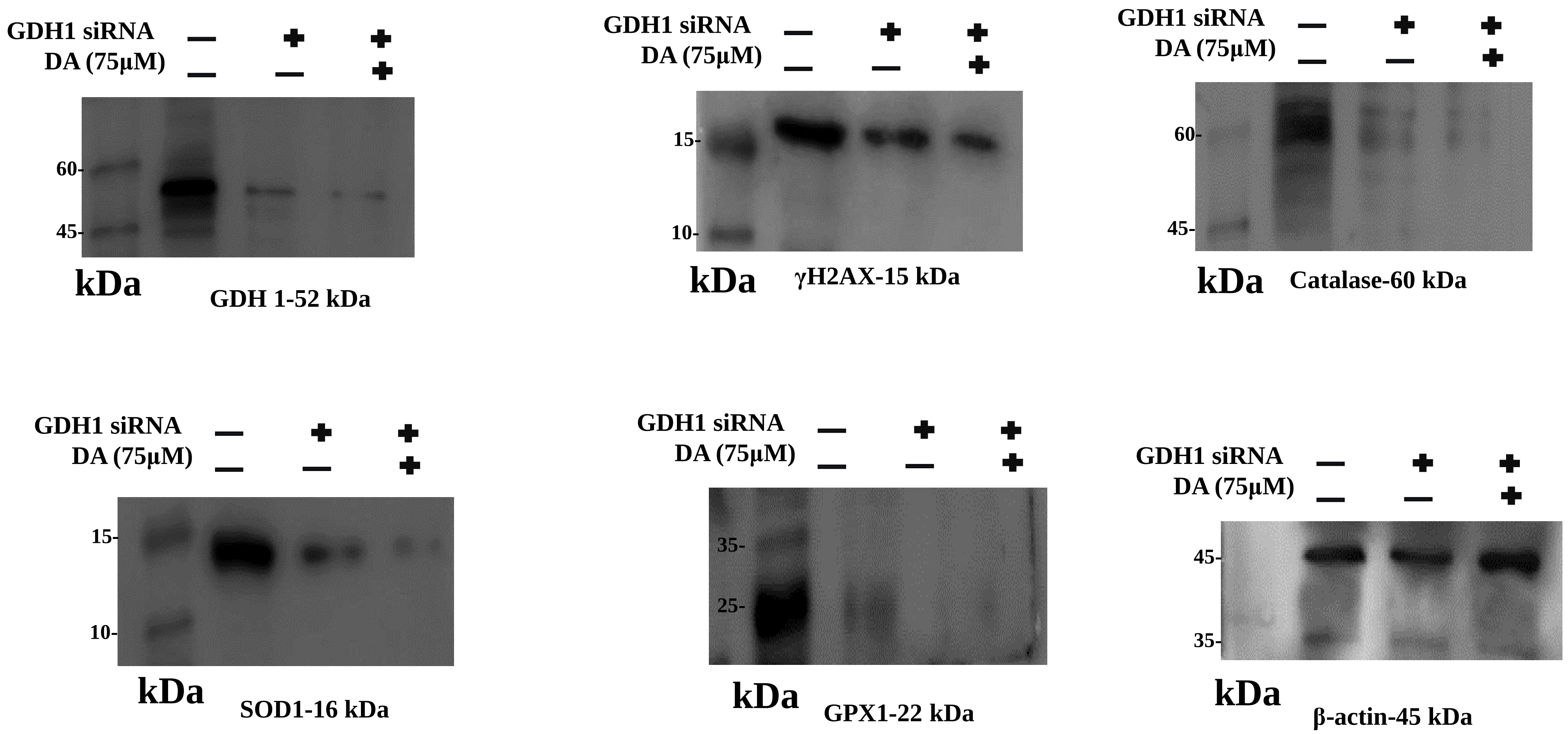

Supplementary Figure S1

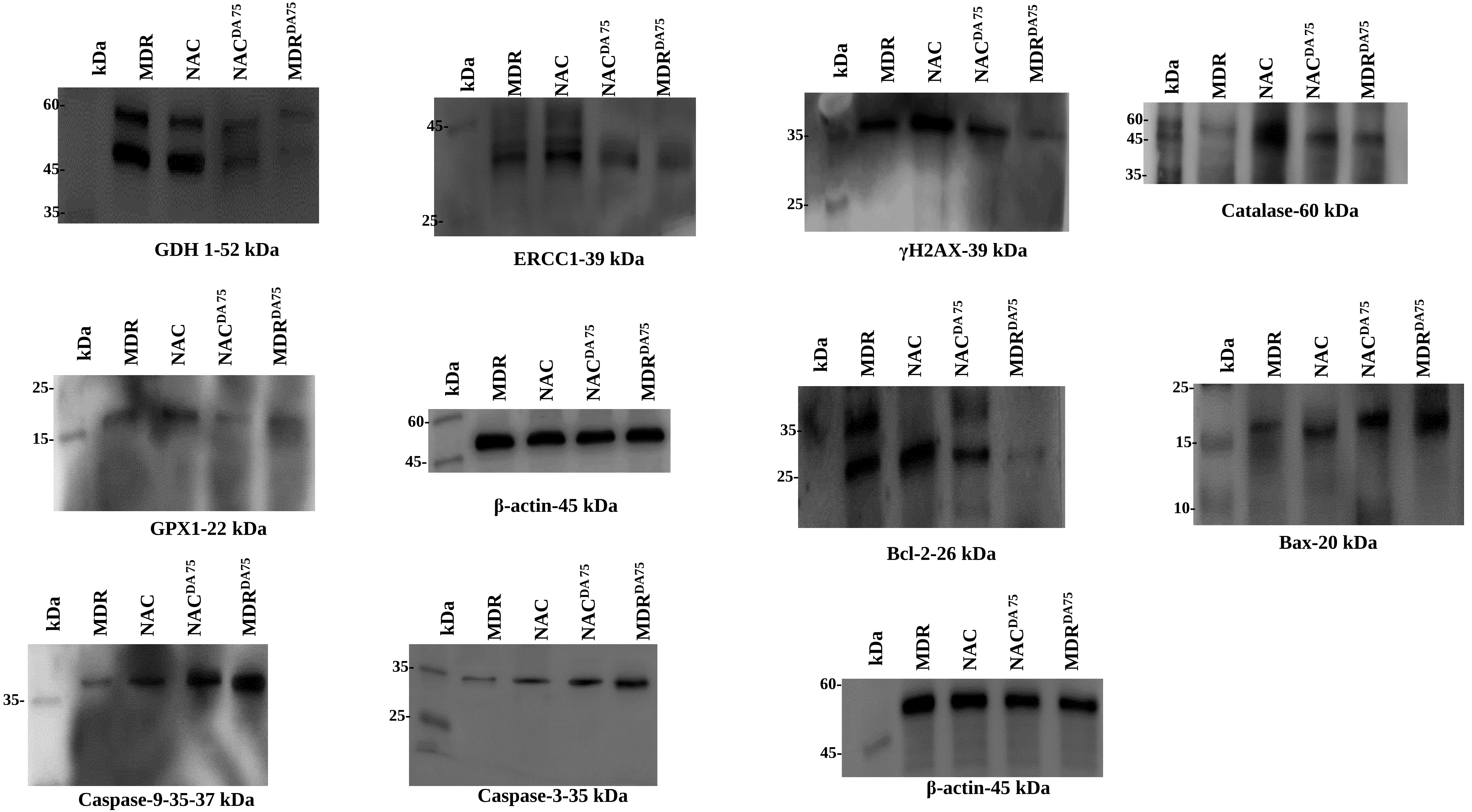

GDH 1-52 kDa

ERCC1-39 kDa

γH2AX-39 kDa

Catalase-60 kDa

GPX1-22 kDa

β-actin-45 kDa

Bcl-2-26 kDa

Bax-20 kDa

Caspase-9-35-37 kDa

Caspase-3-35 kDa

β-actin-45 kDa
