# Supplementary material for: Decursinol Angelate Inhibits Glutamate Dehydrogenase 1 Activity and Induces Intrinsic Apoptosis in MDR-CRC Cells
Source: Cancers (Basel). 2023 Jul 8;15(14):3541. doi: 10.3390/cancers15143541 (PMC10377166; doi:10.3390/cancers15143541)
Supplement: Supplementary file 1 [file cancers-15-03541-s001.zip › cancers-2469817-Supplementary Tables S1-S3 and Figure S1.docx]

**Supplementary Table**.

**Supplementary Table S1.** List of primary antibodies used in the study.

| **S.No.** | **Protein name** | **Company name** | **Molecular weight (kDa)** | **Dilution** | **Host** | **Secondary antibody** |
| --- | --- | --- | --- | --- | --- | --- |
| 1 | GDH1/2  (#12793) | Cell signaling | 52 | 1:100-  1:1000 | Rabbit | Goat anti rabbit-HRP |
| 2 | MDR1  (#12273) | Cell signaling | 130-180 | 1:100-  1:1000 | Rabbit | Goat anti rabbit-HRP |
| 3 | ABCB5  (ABIN390068) | Antibodies online | 89.8 | 1:100-  1:1000 | Mouse | Goat anti mouse-HRP |
| 4 | SOD-1  (sc-11407) | Santa cruz | 23 | 1:100-  1:1000 | Rabbit | Goat anti rabbit-HRP |
|  | SOD-2  (sx-30080) | Santa cruz | 25 | 1:100-  1:1000 | Rabbit | Goat anti rabbit-HRP |
| 5 | Catalase  (sc-50508) | Santa cruz | 64 | 1:100-  1:1000 | Rabbit | Goat anti rabbit-HRP |
| 6 | GPx-1  (sc-22145) | Santa cruz | 23 | 1:100-  1:1000 | Goat | Donkey anti-goat HRP |
| 7 | p53  ( Sc-126) | Santa Cruz | 53 | 1:100-  1:1000 | Rabbit | Goat anti rabbit-HRP |
| 8 | Bax (2772S) | Cell Signaling | 20 | 1:1000 | Rabbit | Goat anti rabbit-HRP |
| 9 | Bcl-2  (ALX-201-701) | ENZO | 26 | 1:1000 | Rabbit | Goat anti rabbit-HRP |
| 10 | Bax  (#2772S) | Cell signaling | 21 | 1:1000 | Rabbit | Goat anti rabbit-HRP |
| 11 | Caspase 9  (Adl-Aap-109) | ENZO | 35,47,48 | 1:1000 | Rabbit | Goat anti rabbit-HRP |
| 12 | Caspase 3  ( BS6428) | Bioworld | 35 | 1:1000  1:5000 | Rabbit | Goat anti rabbit-HRP |
| 13 | Cleaved caspase 3 | Cell signaling | 17,19 | 1:1000 | Rabbit | Goat anti rabbit-HRP |
| 14 | HO-1  (Sc-10789) | Santa Cruz | 32 | 1:100-  1:1000 | Rabbit | Goat anti rabbit-HRP |
| 15 | Nrf2  (BS90969) | Bioworld | 70 | 1:1000  1:5000 | Rabbit | Goat anti rabbit-HRP |
| 16 | GST  (sc-138) | Santa cruz | 28.9 | 1:100-  1:1000 | Mouse | Goat anti mouse-HRP |
| 17 | GR  (sc-133245) | Santa cruz | 52 | 1:100-  1:1000 | Mouse | Goat anti mouse-HRP |
| 18 | ERCC1  (sc-17809) | Santa cruz | 38 | 1:100-  1:1000 | Mouse | Goat anti mouse-HRP |
| 19 | γ-H2aX  (sc517336) | Santa cruz | 15 | 1:100-  1:1000 | Mouse | Goat anti mouse-HRP |
| 20 | β-actin  (#4967) | Cell signaling | 45 | 1:1000 | Rabbit | Goat anti rabbit-HRP |

**Supplementary Table S2**. List of secondary antibodies used in the study.

| **S.No.** | **Name of secondary antibody** | **Company name** | **Dilution** | **Reactivity** |
| --- | --- | --- | --- | --- |
| 1 | Goat anti rabbit-HRP  (NBP2-30348H) | Novus Biologicals (Bethyl) | 1:5000 | Anti-rabbit |
| 2 | Donkey anti goat-HRP  (NBP2-68552) | Novus Biologicals (Bethyl) | 1:5000 | Anti-goat |
| 3 | Goat anti mouse-HRP | Santa cruz | 1:2000-1:10000 | Anti-mouse |
| 4 | Goat anti rabbit-FITC | Santa cruz | 1:100  1:400 | Anti-rabbit |

**Supplementary Table S3**. List of assay kits used in the study.

| **S. No.** | **Name of secondary antibody** | **Company name** |
| --- | --- | --- |
| 1 | ATPase Activity Assay Kit (Colorimetric) | Biovision, Inc. |
| 2 | LDH cytotoxicity Assay Kit  (CAT# KTA1030) | Abbkine |
| 3 | α-Ketoglutarate Colorimetric/Fluorometric Assay Kit | Biovision |
| 4 | Fumarate Colorimetric Assay Kit | Biovision |


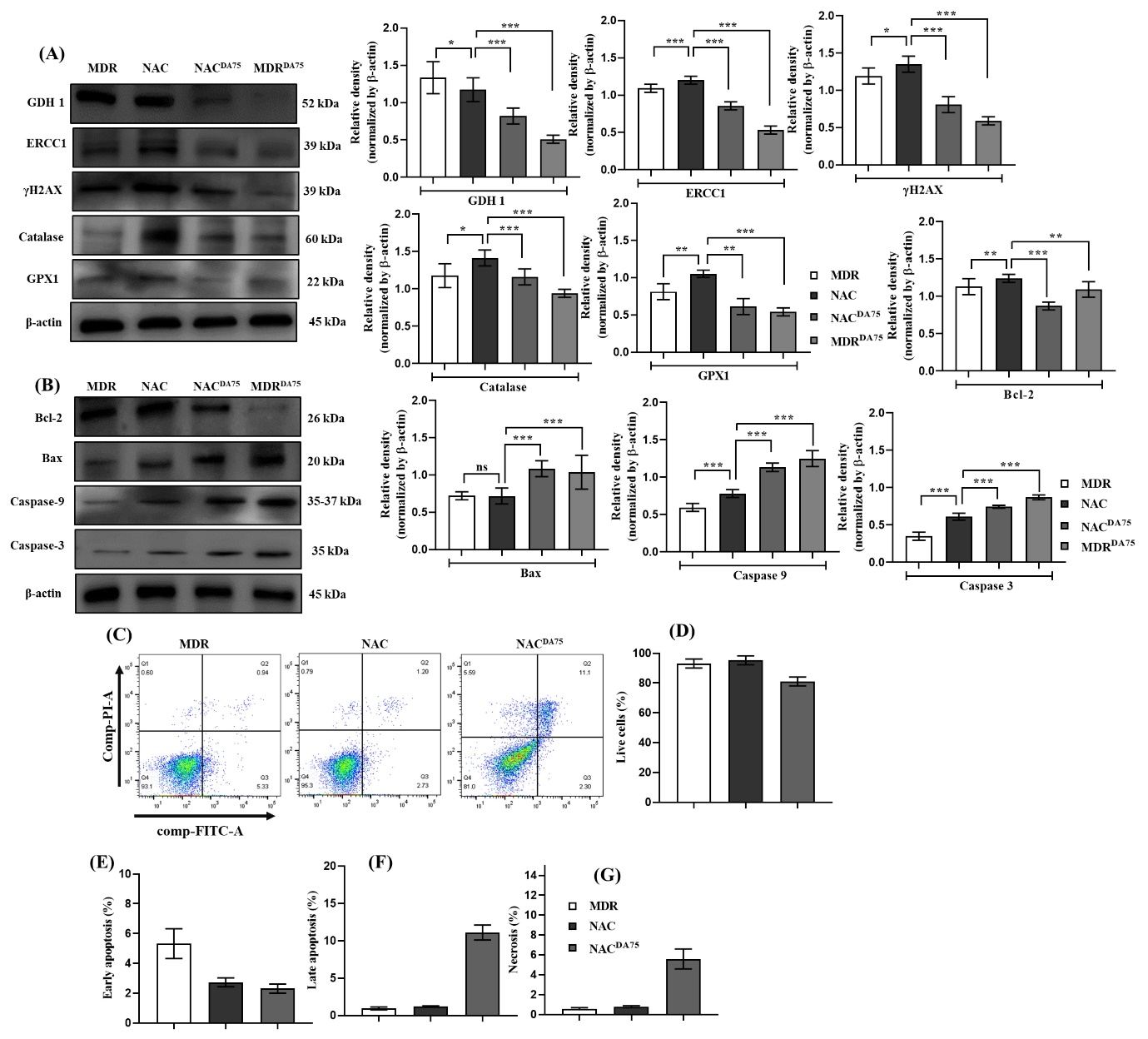


**Supplementary Figure S1.** N-Acetylcysteine (NAC) facilitated reparative property in DA induced damage in MDR cells. (**A**) Western blotting analysis of GDH1, DNA damage repair and antioxidant markers after treatment with NAC (10 mM, 24h) and DA (75 μM, 36 h) in HCT-116^MDR^ cells. (**B**) Western blotting analysis of intrinsic apoptosis markers after treatment with NAC (10 mM, 24h) and DA (75 μM, 36 h) in HCT-116^MDR^ cells (**C**) Annexin V/ propidium iodide flow cytometry evaluation of apoptosis after NAC and DA treatment **(B)** Percentage of live cells after NAC and DA treatment evaluated through flow cytometry (**C**) Percentage of early apoptosis cells after NAC and DA treatment evaluated through flow cytometry (**D**) Percentage of late apoptosis cells after NAC and DA treatment evaluated through flow cytometer **(E**) Percentage of necrotic cells after NAC and DA treatment evaluated through flow cytometry. Densitometry analysis for all the proteins were normalized with β –actin and measured by Image J software. The representative data shown here are the means ± S.D. from three independent experiments where, * *p*< 0.05, ** *p*<0.01, ****p*< 0.001. HCT-116^MDR^ vs NAC, NAC vs all other groups, calculated through ANOVA prism.
